# The molecular language of RNA 5′ ends: guardians of RNA identity and immunity

**DOI:** 10.1261/rna.079942.124

**Published:** 2024-04

**Authors:** Rodolfo Gamaliel Avila-Bonilla, Sara Macias

**Affiliations:** Institute of Immunology and Infection Research, School of Biological Sciences, University of Edinburgh, EH9 3FL Edinburgh, United Kingdom

**Keywords:** 5′ cap, m7G, IFIT, RIG-I, capping, CMTR1, CMTR2, type I interferon, antiviral

## Abstract

RNA caps are deposited at the 5′ end of RNA polymerase II transcripts. This modification regulates several steps of gene expression, in addition to marking transcripts as self to enable the innate immune system to distinguish them from uncapped foreign RNAs, including those derived from viruses. Specialized immune sensors, such as RIG-I and IFITs, trigger antiviral responses upon recognition of uncapped cytoplasmic transcripts. Interestingly, uncapped transcripts can also be produced by mammalian hosts. For instance, 5′-triphosphate RNAs are generated by RNA polymerase III transcription, including tRNAs, Alu RNAs, or vault RNAs. These RNAs have emerged as key players of innate immunity, as they can be recognized by the antiviral sensors. Mechanisms that regulate the presence of 5′-triphosphates, such as 5′-end dephosphorylation or RNA editing, prevent immune recognition of endogenous RNAs and excessive inflammation. Here, we provide a comprehensive overview of the complexity of RNA cap structures and 5′-triphosphate RNAs, highlighting their roles in transcript identity, immune surveillance, and disease.

## INTRODUCTION

As part of the innate immune response to pathogens, mammalian cells express pathogen recognition receptors (PRRs), which are capable of recognizing specific molecular traits in infectious agents, such as virus-derived nucleic acids. Although viral and host nucleic acids are composed of the same base nucleotides, they differ in their secondary structure, post-transcriptional modifications, and subcellular localization. The unique characteristics of viral transcripts are exploited by the innate immune system to appropriately discriminate self from nonself nucleic acids. For instance, the presence of viral cytoplasmic DNA is sensed by the innate immune sensor cGAS, activating the type I interferon (IFN) response. The accumulation of double-stranded RNAs (dsRNAs) in the cytoplasm of infected cells can also trigger the type I IFN response by the RIG-I-like receptors (RLRs), in addition to activating the host translational shutoff by the dsRNA-activated protein kinase R (PKR). The presence of specific epigenetic modifications in the host's nucleic acids provides an additional layer of discrimination. However, viruses can hijack these modifications to prevent being recognized by the innate immune system (Han and Xu 2023). For RNA alone, there are more than 170 different types of modifications described (Wiener and Schwartz 2021). An important modification is the addition of the 5′ cap structure at the 5′ end during RNA polymerase II transcription of eukaryotic RNAs. Caps are added to an array of different RNA types, including pre-messenger RNAs (pre-mRNAs), long noncoding RNAs (lncRNAs), and primary microRNAs (pri-miRNAs), among others (Pelletier et al. 2021). The interaction of the cap structure with cap-binding or interacting proteins is critical to control key aspects of the gene expression pathway, including pre-mRNA processing, mRNA stability, nuclear export, and mRNA translation. In addition, this modification is critical in safeguarding endogenous RNAs from recognition by the innate immune sensing pathway (Galloway and Cowling 2019). The absence of cap structures can result in the activation of specific innate immune sensors. These sensors include RIG-I (retinoic acid-inducible gene I) and the IFIT (interferon-induced protein with tetratricopeptide repeats) family of proteins, which initiate an antiviral innate immune response (Leung and Amarasinghe 2016; Liang et al. 2023).

## THE MAMMALIAN 5′ CAP STRUCTURE

**Figure 1.**
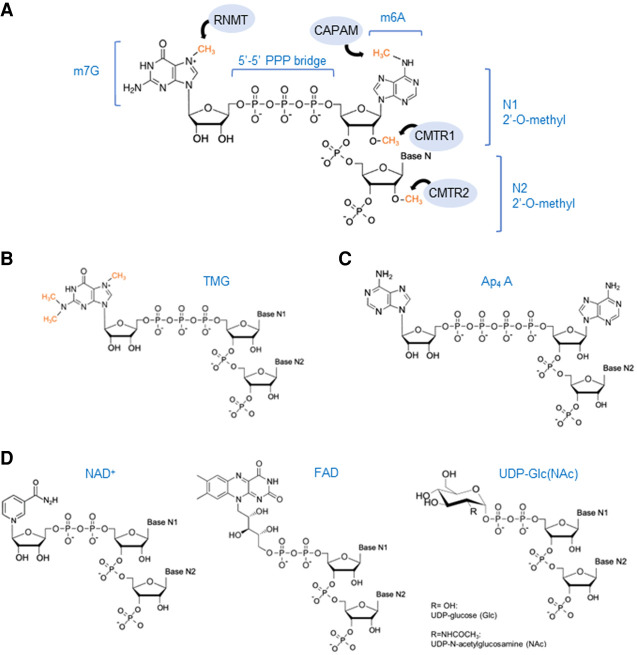
5′ RNA cap structures. (*A*) Structure of m7G cap linked to the 5′-5′ triphosphate bridge (or cap 0) and m6A methylation if the first transcribed nucleotide is an adenosine. Additionally, both the first (N1, cap 1) and the second (N2, cap 2) can be 2′-*O*-methylated. Enzymes involved in each of these modifications are illustrated in blue circles and methyl groups are marked in orange. (RNTM) RNA guanine-7 methyltransferase, (CAPAM) cap-specific adenosine N6-methyltransferase, and (CMTR) cap 2′-*O*-methyltransferase 1 and 2. (*B*) Tri-methylguanosine (TMG) cap structure. Methyl groups at the N2 position on the cap 0 structure are marked in orange. (*C*) Structure of the noncanonical RNA cap, diadenosine tetraphosphate (Ap_4_ A). (*D*) Metabolite-derived cap structures. (*Left* to *right*) Nicotinamide adenine dinucleotide (NAD) caps can exist in the oxidized form, as NAD^+^ (shown), or in the reduced form, NADH. Flavin adenine dinucleotide (FAD) caps structures. UDP-glucose-derived caps include uridine diphosphate-glucose (UDP-Glc) and UDP-*N*-acetylglucosamine (UDP-GlcNAc).

Typically, the mammalian 5′ cap structure consists of a guanosine nucleotide methylated at its N7 position, linked to the 5′ end of the mRNA strand via a 5′-5′ triphosphate linkage. This cap configuration is referred to as the m7G cap or cap 0. mRNAs can also be 2′-*O*-methylated at the first (N1) and second (N2) transcribed nucleotides. These modified RNAs are denoted as cap 1, when only N1 is methylated, and cap 2, when both N1 and N2 are methylated. When the first transcribed nucleotide is an adenosine, this can also be N6-methylated (m6Am) (Shatkin 1976). These cap structures are summarized in Figure 1A.

In mammals, capping takes place after transcription of the first 20–30 nt, and it is initiated by the bifunctional mRNA capping enzyme, RNGTT, which contains both the RNA 5′-triphosphate monophosphatase and the mRNA guanylyltransferase (GTase) activities. This enzyme converts the 5′-triphosphate (5′-PPP) end of the first transcribed nucleotide to diphosphate, followed by the transfer of an inverted guanosine (GMP) by the RNA GTase activity. Next, the RNA guanine-7 methyltransferase (RNMT) in complex with the RNMP-activating mini-protein (RAM) methylates the N7 position of the guanosine, resulting in m7G or cap 0 structure (Yue et al. 1997; Ho et al. 1998; Gonatopoulos-Pournatzis et al. 2011; Ramanathan et al. 2016). These enzymes can also recap mRNAs in the cytoplasm (Schoenberg 2021). In contrast to yeast, where mRNAs exclusively exhibit the cap 0 structure, the cap configuration in higher eukaryotes can undergo further modifications, which involve methylation at the 2′-*O*-ribose position of the initial (N1) and second (N2) nucleotides. The first 2′-*O*-methylation occurs at the N1 residue, and it is guided by the cap methyltransferase 1 (CMTR1) in the nucleus. This modification happens cotranscriptionally, as this enzyme is recruited to the nascent RNA by RNA polymerase II (Langberg and Moss 1981; Haline-Vaz et al. 2008; Bélanger et al. 2010; Inesta-Vaquera et al. 2018). By binding to transcription start sites (TSSs) CMTR1 can also promote RNA transcription. In addition, N1-modified RNAs show increased stability and translation (Picard-Jean et al. 2018; Sikorski et al. 2020; Williams et al. 2020; Liang et al. 2022). Methylation of the second transcribed residue (N2) takes place in the cytoplasm, and it is guided by the cap methyltransferase 2 (CMTR2) (Werner et al. 2011). Although the function of N2 methylation is less clear, it has also been associated with increased mRNA stability and translation. In terms of mRNA stability, both N1 and N2 methylations protect RNAs from degradation by DXO, a decapping exoribonuclease capable of hydrolyzing the bonds between the first and second transcribed nucleotide to degrade the RNA in a 5′- to 3′-direction (Picard-Jean et al. 2018; Drazkowska et al. 2022). In addition to 2′-*O*-methylation, when the first transcribed nucleotide is an adenosine, further modification by methylation in position N6 (m6A) by the cap-specific adenosine N6-methyltransferase PCIF1/CAPAM can occur. This modification is different from the internal m6A methylation (Wei et al. 1975; Keith et al. 1978; Akichika et al. 2019; Boulias et al. 2019; Sendinc et al. 2019; Sun et al. 2019; Anreiter et al. 2023). CMTR1 and PCIF1/CAPAM methylate the N1 residue in a sequential manner, with m6A methylation occurring after 2′-*O*-methylation (Boulias et al. 2019; Yu et al. 2022). The cooperation of these two activities leads to the formation of a highly distinctive cap structure known as cap-specific N6-methyladenosine (m6Am), which is highly abundant within mRNAs (Sun et al. 2019; Galloway et al. 2020). m6Am mRNAs exhibit enhanced stability by being resistant to the decapping enzyme DCP2, in addition to increased translation (Dunckley and Parker, 1999; Mauer et al. 2017; Akichika et al. 2019; Boulias et al. 2019; Sikorski et al. 2020).

Mass spectrometry analyses have provided critical information on the relative abundance of these modifications. For instance, in mouse tissues, most caps contain an N7-methylated residue and 2′-*O*-methylation in N1, with only 2%–5% lacking these. In addition, there are more caps containing N6-methylated adenosines than not (Galloway et al. 2020). CLAM-Cap-seq, a method used to quantify cap 2, revealed that most long-lived mRNAs are N2-methylated through a slow conversion of mRNAs from cap 1 to cap 2 in the cytosol (Despic and Jaffrey, 2023).

Other classes of caps include the TMG cap, which consists of the addition of two methyl groups at the N2 position of the cap 0 structure of small nuclear RNAs (U1, U2, U4, and U5), small nucleolar RNAs and telomerase RNA (Fig. 1B; Mattaj 1986; Buemi et al. 2022). This modification is important for the nuclear localization of these noncoding RNAs (ncRNAs) (Moreno et al. 2009).

More recently, noncanonical caps have been described. These include Ap4A (diadenosine tetraphosphate) caps, NAD (nicotinamide adenine dinucleotide), either in the oxidized (NAD^+^) or reduced form (NADH), FAD (flavin adenine dinucleotide) and UDP-glucose-derived caps, including UDP-Glc/GlcNAc (uridine diphosphate glucose or *N*-acetylglucosamine) caps (Fig. 1C,D). These types of caps are present in both prokaryotes and eukaryotes, but also viruses. For instance, hepatitis C (HCV) viral transcripts are capped with FAD (Cahová et al. 2015; Jiao et al. 2017; Wang et al. 2019; Sherwood et al. 2023; František Potužník et al. 2024) The biosynthesis and function of these less conventional caps have been recently reviewed in Mattay (2022).

## THE 5′ END OF RNAs AS A HUB FOR IDENTITY AND INNATE IMMUNE RECOGNITION

**FIGURE 2.**
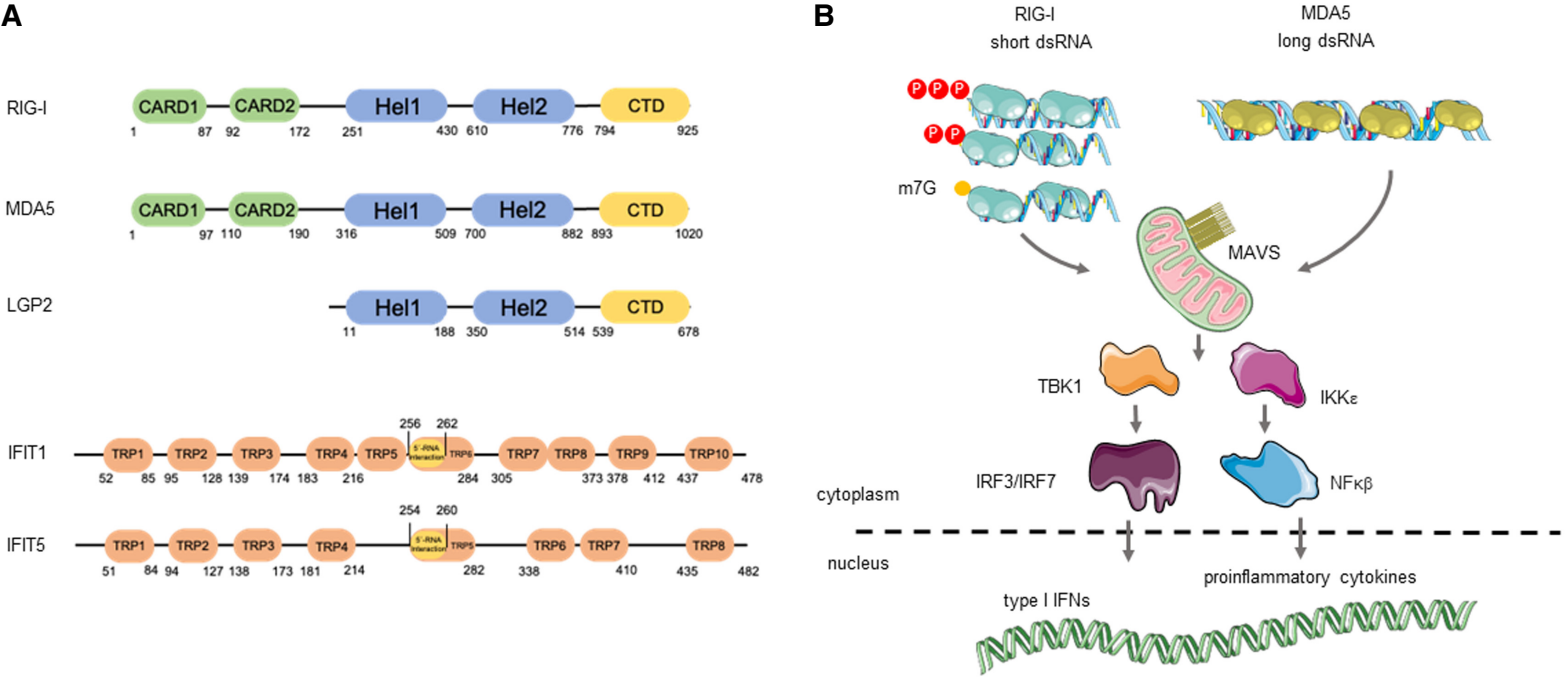
Innate immune sensors for 5′-end RNA recognition. (*A*) Functional domains of the human RLR-pathway receptors, RIG-I, MDA5, and LGP2 (*top*), and IFIT members, IFIT1 and IFIT5 (*bottom*). (CARD) Caspase activation and recruitment domains; (Hel) helicase domain; (CTD) C-terminal domain; (TRP) tetratricopeptide repeat motif. Numbers indicate residue position. (*B*) The RIG-I-like receptor RIG-I recognizes single-stranded RNA (ssRNA) or dsRNA containing 5′-PPP, or 5′-diphosphate ends (5′-PP). RIG-I can also bind m7G-RNAs that are unmethylated in positions N1 and N2. The other major RLR receptor MDA5 is specialized in recognizing long dsRNAs. Upon binding to viral RNAs, RIG-I and MDA5 signal through the mitochondrial-associated protein, MAVS. Next, the kinases TBK1 and IKKe are activated, promoting the nuclear translocation of the IRF3/7 and NFkB transcription factors, which drive expression of type I IFNs and proinflammatory cytokines.

In mammalian cells, several PRRs, including RIG-I and the IFIT family of proteins, can recognize the nature of the 5′ end of cytoplasmic RNAs and discriminate between the host's and the virus’ nucleic acids to appropriately activate innate immune responses (Fig. 2). The mechanisms of action of these proteins are described below.

### RIG-I

RIG-I (retinoic acid inducible gene-I, *DDX58*) belongs to the RIG-I-like family of receptors (RLRs), together with MDA5 (melanoma differentiation-associated protein 5, *IFIH1*) and LGP2 (laboratory of genetics and physiology 2, *DHX58*). These are all cytoplasmic localized nucleic acid sensors which can recognize virus-derived dsRNA of different lengths. While RIG-I and MDA5 are structurally similar and can both signal to activate the IFN response, LGP2 seems to be signaling incompetent and to regulate both the function of RIG-I and MDA5 (Yoneyama et al. 2005; Bruns et al. 2014; Thoresen et al. 2021). Both RIG-I and MDA5 contain two N-terminal caspase activation and recruitment domains (CARDs), a central helicase domain, and a C-terminal domain (CTD). LGP2 lacks the CARD domains, which are important for signal transduction (Fig. 2A). Upon dsRNA recognition, RLRs oligomerize on the RNA, exposing their CARD domains to interact with the CARDs from the mitochondrial antiviral signaling protein (MAVS). Next, MAVS activates the TANK-binding (TBK1) and IKK kinases resulting in the nuclear translocation of both IRF3/7 and NFkB transcription factors. These transcription factors are responsible for inducing the expression of type I IFNs and proinflammatory cytokines (Fig. 2B). Secreted IFNs stimulate the expression of hundreds of IFN-stimulated genes (ISGs), including RIG-I, MDA5, and IFN-induced protein with tetratricopeptide 1 (IFIT1). This second signaling cascade amplifies the IFN response, and it is responsible for establishing an antiviral cellular state in both the infected and neighboring cells, preventing viral replication and dissemination (for reviews, see Watson et al. 2019; Rehwinkel and Gack 2020).

RIG-I is also known to play a role in 5′-end RNA recognition. RIG-I was originally described to recognize blunt-ended dsRNAs containing either 5′-di- (5′-PP) or 5′-triphosphates (5′-PPP). In contrast, 5′-monophosphates (5′-P) completely abrogate RIG-I binding, even more efficiently than 5′-OH ends. Uncapped dsRNAs typically accumulate during the replication of ssRNA (+) or (−) strand viruses (Hornung et al. 2006; Pichlmair et al. 2006; Rehwinkel et al. 2010; Wang et al. 2010; Goubau et al. 2014; Ren et al. 2019). In addition to 5′-PPP or 5′-PP dsRNAs, RIG-I can also bind capped (m7G) RNAs that are not methylated in position N1, or cap 0 RNAs (Schuberth-Wagner et al. 2015; Devarkar et al. 2016). N1-methylation dramatically reduces RIG-I affinity, as the RNA binding pocket of RIG-I cannot accommodate N1-methylated RNAs. On the other hand, N2 methylation on its own does not fully prevent RIG-I recognition, but the cap 2 (N1 + N2) modification does, more efficiently than only N1 (Wang et al. 2010; Schuberth-Wagner et al. 2015; Devarkar et al. 2016; Despic and Jaffrey 2023). The cap-specific N6-methyladenosine (m6Am) modification may also have a role in preventing innate immune sensing. Transcripts derived from vesicular stomatitis virus (VSV) and Rabies virus are methylated by PCIF1/CAPAM, suggesting a role for this modification in innate immune evasion (Tartell et al. 2021). Although bulky metabolite-modified caps (NAD/FAD) have been proposed to stimulate RIG-I ATPase activity to levels similar of 5′-PPP RNAs, the presence of FAD-caps in HCV transcripts is associated with decreased RIG-I recognition and innate immune evasion (Schweibenz et al. 2023; Sherwood et al. 2023). For a complete summary, see Table 1.

**TABLE 1.** 5′ end of RNAs recognized by antiviral innate immune sensors

RNA 5′ ends	RIG-I	IFIT1	IFIT5
5′-P	−	−	++
5′-PPP/PP	++	++	++
m7G (cap 0)	++	++	+
m7G N1 (cap 1)	−	+	−
m7G N2 (cap 2)	−	−	−

(++) Interaction.

(+) Weak interaction.

(−) No interaction.

### IFITs

Another important sensor for uncapped RNAs is IFIT1 (IFN-induced protein with tetratricopeptide 1, *ISG56*), which belongs to the IFIT family (Fig. 2B). In humans, this family is composed of IFIT1 (*ISG56*), IFIT2 (*ISG54*), IFIT3 (*ISG60*), and IFIT5 (*ISG58*). *IFIT* genes are arranged in a gene cluster in Chromosome 10, which also contains an additional member, *IFIT1B*. Compared to the other IFIT family members, *IFIT1B* is the only one lacking the IFN-stimulated response element (ISRE) in its promoter, indicating that it is not an ISG (Fensterl and Sen 2011; Daugherty et al. 2016). Regarding their antiviral activity, IFIT proteins have been shown to interfere with viral mRNA translation. IFIT1 was initially found to bind eIF3 and impair cap-dependent translation of reporter genes, both in vitro and in cells, as well as inducing a general inhibition of all protein synthesis upon overexpression (Guo et al. 2000; Hui et al. 2003). More recent work indicates that IFIT1 does not directly associate with the translation machinery, but instead competes with other 5′ cap recognition proteins, including important factors for the initiation of mRNA translation, such as eIF4E and eIF4F. This function is also facilitated by its interaction with IFIT2 and IFIT3 (Pichlmair et al. 2011; Habjan et al. 2013; Kumar et al. 2014; Fleith et al. 2018). Formation of this complex is important for antiviral defense, as independent knockdown of IFIT1, 2, or 3 results in increased viral replication of VSV and influenza A virus (IAV) (Pichlmair et al. 2011; Stawowczyk et al. 2011). Mears and Sweeney compiled a list of viruses affected by mouse or human IFIT1 in their review (Mears and Sweeney 2018). The interaction between IFIT1 and IFIT3 or the dimer IFIT2:3 is also important to enhance cap 0-RNA binding affinity and promote IFIT1 protein stabilization (Fleith et al. 2018; Johnson et al. 2018). In addition to IFIT1, IFIT2 can also bind RNA containing AU-rich sequences, but without obvious preference for the type of 5′ end (Yang et al. 2012).

In terms of 5′-end RNA recognition, both human IFIT1 and IFIT5 are capable of binding 5′-PPP RNAs (Pichlmair et al. 2011; Abbas et al. 2013), although IFIT5 binds with stronger affinity (Kumar et al. 2014). IFIT5 is shown to bind both to 5′-P and 5′-PPP with similar affinities, but not capped RNAs (Katibah et al. 2014). Instead, IFIT1 is the only member that binds proficiently to capped RNAs lacking methylation of the first and second transcribed residues (N1 and N2), but also RNAs lacking N7-methylation of the cap, suggesting that it recognizes unmethylated RNAs (Habjan et al. 2013; Kumar et al. 2014, Abbas et al. 2017). In agreement, the combination of both N1 and N2 methylation is the most efficient in preventing IFIT1 recognition (Abbas et al. 2017). Therefore, IFIT1 can block the in vitro translation ability of RNAs containing an unmethylated cap guanosine, lacking N7-methylation, or cap 0 mRNAs, lacking N1 2′-*O*-methylation. This effect is partially abrogated by N1-methylation (Young et al. 2016). Structural comparisons of IFIT1 and IFIT5 confirm their different substrate preferences, with IFIT1 recognizing capped but unmethylated RNAs, while IFIT5 binding 5′-PPP RNAs (Abbas et al. 2013, 2017). It is important to note that the functions of human and mouse IFIT members are not fully conserved. For instance, mouse *Ifit1* can only act on cap 0 mRNAs, while human *IFIT1* can engage with both cap 0 and cap 1 mRNAs. Rodents have also lost the *IFIT5* gene (Daugherty et al. 2016).

Regarding metabolite-derived caps, IFIT1 binds poorly to NAD^+^ or NADH-RNAs, while IFIT5 bound stronger to this type of RNAs, especially NADH-RNAs, with comparable affinity to 5′-PPP RNAs (Miedziak et al. 2020). For a complete summary, see Table 1.

## FUNCTIONS OF THE 5′-END SENSORS AND MODIFYING ENZYMES BEYOND VIRAL RESTRICTION

In mammals, most of the ncRNAs transcribed by the RNA polymerase III bear a 5′-PPP end, suggesting that in certain settings these transcripts could be aberrantly recognized by some innate immune sensors as pathogenic. RNA polymerase III transcribed RNAs include, 5S rRNA, tRNAs, 7SL RNAs, U6 snRNA, RNase P and MRP RNAs, small nucleolar RNAs, 7SK RNA, Alu RNAs, vault RNAs (vRNAs), and Y RNAs (Dieci et al. 2007; Cai et al. 2022). In addition to the host's genes, RNA polymerase III can also transcribe the genome of DNA viruses, thus generating 5′-PPP RNAs that can be recognized by RIG-I, thus amplifying the innate immune response (Jarrous and Rouvinski 2021; Naesens et al. 2023). RNA polymerase III transcripts lack a capping mechanism, therefore host 5′-PPP RNAs need to be further processed to avoid RIG-I recognition. One mechanism is driven by the dual-specificity phosphatase 11, DUSP11. This is an RNA triphosphatase that converts 5′-PPP to 5′-P both from viral transcripts, such as viral-encoded miRNAs, but also host transcripts, including vRNAs and Alu transcripts (Deshpande et al. 1999; Burke et al. 2014, 2016; Burke and Sullivan 2017). In the absence of DUSP11, a type I IFN signature is observed without infection. This is presumably due to the accumulation of 5′-PPP RNAs, which can be recognized by RIG-I to induce the IFN response (Choi et al. 2020). Similarly, during Kaposi's sarcoma-associated herpesvirus (KSHV) lytic reactivation, the levels of DUSP11 are reduced, resulting in recognition of 5′-PPP vRNAs and IFN activation (Zhao et al. 2018). Endogenous RNA polymerase III transcripts that have been shown to be recognized by RIG-I include: 5S rRNA pseudogenes, 7SL RNA, Alus, U6 snRNA, 7SK RNA, and Y-RNA, among others (Burke et al. 2016; Nabet et al. 2017; Chiang et al. 2018; Wu et al. 2018; Zhao et al. 2018; Vabret et al. 2022; Zhang et al. 2022; Naesens et al. 2023). In addition, RIG-I has been involved in circular RNA (circRNA) recognition, although contradictory findings have been reported. CircRNAs are ncRNAs formed by a process known as back-splicing, and although their function is still unclear, they have been proposed to act as RNA or protein sponges (Kristensen et al. 2019). RIG-I has been proposed to discriminate between endogenous circRNAs originated from mammalian introns and foreign circRNAs, independently of 5′-PPP formation (Chen et al. 2017, 2019). However, an independent report suggests that RNA circularization prevents innate immune recognition by RIG-I and concludes that innate immune activation by circularized RNAs can be derived from impurities generated during in vitro synthesis (Wesselhoeft et al. 2019). RNA circularization has also been suggested to be exploited by viruses to evade the innate immune response, including the hepatitis D virus (Kos et al. 1986; Jung et al. 2020).

In humans, missense, gain-of-function mutations of the RIG-I gene (*DDX58*) are associated with constitutive IFN signaling causing the atypical Singleton–Merten syndrome (Jang et al. 2015; Ferreira et al. 2019; Prasov et al. 2022). The disease manifestations include glaucoma, calcification of the aorta, and dental and skin issues, such as psoriasis. Mechanistically, those gain-of-function mutations lead to a constitutively activated RIG-I form, unable to dissociate from endogenous self-RNAs, resulting in constant IFN signaling (Devarkar et al. 2018; Lässig et al. 2018; Lei et al. 2022). Which endogenous RNAs drive IFN activation in the context of this syndrome are still unknown.

Among the IFIT family, IFIT5 can also recognize cellular RNAs. IFIT5 is the only member that cannot associate with other IFIT family members (Pichlmair et al. 2011; Katibah et al. 2013). IFIT5 can also bind to cellular RNAs harboring 5′-P or 5′-PPP RNAs, including precursor and mature tRNAs, tRNA pseudogenes, and 5S rRNA (Katibah et al. 2013, 2014). In agreement with IFIT5 preference in binding, precursor tRNAs are initially transcribed as 5′-PPP RNAs, but become monophosphorylated after processing by RNase P (Betat et al. 2014). Previously, IFIT5 had also been shown to bind the Y RNA, Y5 (Hogg and Collins 2007).

In addition to 5′-end recognition and modification, IFIT proteins and the RNA methyltransferases have additional roles in the innate immune response. For instance, human IFIT5 negatively regulates the type I IFN response in HEK293T cells by preventing IRF3 activation (Zhang et al. 2021). In contrast, IFIT1 has been shown to be required for optimal expression of the type I IFN, *IFNB1*, and ISGs in response to the viral mimic, poly(I:C), but also to the lipopolysaccharide (LPS), suggesting that it may also have a role in antibacterial defence. This function seems to be carried out by a small fraction of IFIT1 that localizes in the nucleus and that is responsible for perturbing the transcriptional silencer, Sin3A-HDAC, allowing *IFNB1* transcription (John et al. 2018). Similarly, IFIT1 has been shown to be responsible for the efficient production of *CXCL10*, a chemokine produced in response to poly(I:C) (Shiratori et al. 2020). In the context of KSHV lytic reactivation, IFIT1 has also been shown to bind viral RNAs as well as cellular mRNAs, including *GAPDH*, mitochondrial RNAs, snRNAs, and to a lesser extent, snoRNAs (Li and Swaminathan 2019). In this context, IFIT1 was also found to be important for robust IFN-β production. In addition, IFIT1 has been show to coimmunoprecipitate with the ORF1p from the human LINE-1 retrotransposon. Despite this interaction, IFIT1 was not shown to affect the levels of LINE-1 ORF1p or LINE-1 RNA, and it only mildly affected its retrotransposition rate (Luqman-Fatah et al. 2023).

Similar to IFITs, CMTR1 has been shown to be required for efficient translation of ISG mRNAs. In the absence of CMTR1, and therefore N1 2′-*O*-methylation, IFIT1 can bind to ISGs, leading to reduced translation of these mRNAs. In agreement, ISG protein levels are rescued after *IFIT1* knockdown in CMTR1-depleted cells. All these suggest that induction of CMTR1, which is an ISG itself, is required to avoid innate immune recognition of unmethylated endogenous RNAs by IFIT1 (Williams et al. 2020). CMTR1 has also been shown to be required for suppressing RIG-I recognition of cellular RNAs during homeostasis. Knockdown of CMTR1 in primary human fibroblasts or A549 cells results in the induction of the type I IFN response. IFN activation is counteracted by the depletion of RIG-I, suggesting that RIG-I can recognize both cellular capped (cap 0) and uncapped (5′-PPP) RNAs (Schuberth-Wagner et al. 2015). Structural analyses also support a role of RIG-I in recognizing both 5′-PPP and cap 0 dsRNAs (Devarkar et al. 2016). Similarly, CMTR1-KO A549 cells display an increased IFN signature in the context of IAV (PR8) infection, and this is also mediated by RIG-I signaling; however, no IFN signature was observed in the resting state (Li et al. 2020). In mice, deletion of CMTR1 in the adult liver resulted in the up-regulation of several ISGs, including IFIT1, possibly by sensing of endogenous transcripts. CMTR1 was also found to be essential for mouse embryonic development without activating the type I IFN response, as the ability to produce IFNs is inactive during the early stage of development (Gázquez-Gutiérrez et al. 2021; Dohnalkova et al. 2023; Mueller et al. 2024). In addition to CMTR1, the absence of CMTR2 in HEK293T also results in activation of the type I IFN response in a RIG-I-dependent manner (Despic and Jeffrey 2023). Interestingly, CMTR2 is also essential for mouse embryonic development (Dohnalkova et al. 2023).

## SUMMARY AND OUTLOOK

All these results suggest that capping and methylation of the host's mammalian transcripts are required to avoid self-recognition. In the absence of these modifications, the innate immune system recognizes endogenous RNAs as foreign and activates antiviral defences inappropriately. In the future, it will be interesting to investigate if recognition of endogenous transcripts by RIG-I or IFITs is an unavoidable mistake, or if instead, this can have a functional and positive role for the cell. For instance, the RLR family member MDA5 triggers the innate immune response upon binding endogenous RNAs to promote hematopoietic stem cell proliferation and replenish the bone marrow after chemotherapy (Clapes et al. 2021). Also, recognition of cellular RNA Pol III-derived transcripts by RIG-I during infection is important to potentiate the innate immune response against viruses (Naesens et al. 2023). IFITs have been involved in both cancer progression and metastasis, suggesting that they may also contribute to the normal functioning of the cell and have a role outside the antiviral response (Pidugu et al. 2019). Further research on these antiviral factors will help us understand if cells have co-opted the recognition of endogenous cellular RNAs as an essential activity for maintaining homeostatic function.

Understanding in more detail the mechanisms for RNA-based innate immune sensing is critical to develop better methods for mRNA-based therapy, including mRNA vaccination or expression of therapeutic proteins (Ramanathan et al. 2016). Investigating viruses and the function of viral proteins will be key to better understand the innate immune response to infection. Viruses are in constant evolution to counteract the action of the multiple immune sensors and antiviral proteins. In terms of capping, viruses have developed mechanisms to cap and methylate their own RNAs in the cytoplasm, independently from the host's capping machinery. The NS5 protein from flaviviruses guides guanine N7 and 2′-*O*-methylations to avoid innate immune recognition (Zhou et al. 2007; Daffis et al. 2010), and IAV uses cap-snatching, a method which involves stealing 5′ ends of host's RNAs containing caps (Plotch et al. 1981) This process results in the generation of new proteins from the fusion of host and viral mRNAs (Plotch et al. 1981; Ho et al. 2020). All these examples highlight the constant arms race between viruses and their host's immune defences, and the importance of understanding 5′-end RNA innate immune recognition.
